# Notes and News

**Published:** 1887-10-22

**Authors:** 


### NOTES AND NEWS.

COLLECTED AND COMMUNICATED.

OWING to great pressure on our space, due to the interest created by the Pension Fund meeting, we are compelled to hold over several papers and letters till next week.

THE Marquis of Carmarthen, M.P., has accepted the office of president of the London Skin Hospital, Cranbourne Street, Leicester Square.

SIR WILLIAM C. BROOKS, Bart., M.P., Forest of Glen Jana, Aboyne, N.B., has sent a handsome present of venison of red deer for the inmates of the Cottage Hospital, Congleton.

DR. FORBES WINSLOW and Dr. G. Herschell have been appointed additional physicians to the WEST-END HOSPITAL for Diseases of the Nervous System, Paralysis, and Epilepsy.

BIRMINGHAM is said by the *Birmingham Mail* to be suffering from an epidemic of "appeals" from its various charities. Perhaps if the Birmingham people were more liberal the epidemic might soon be stamped out.

A NEW wing is to be added to the Royal South Hants Infirmary in commemoration of Her Majesty's Jubilee. The total cost will be £1,575, of which the greater part is already in hand, as the result of the Jubilee efforts.

THE *Oswestry Advertiser* says :—"We publish to-day several letters on the Oswestry Cottage Hospital, and a copy of resolutions in which the committee exonerate the staff from blame in connection with two cases which have caused great excitement in the town. Want of space prevents us from considering the matter at any length now, but the committee ought to be informed that the very strongest feeling exists on the subject, and that in the opinion of the public a change in the rules is imperatively demanded. The refusal of admission to the poor fellow Williams, unfortunately followed by the removal of Mrs. McNicholas, has caused quite a shock of pained surprise, and we who have constantly commended the hospital to the generous support of our readers, sincerely trust that we shall soon have the great satisfaction of publishing such a statement as will quiet the public mind."

THERE has been no falling off in the Hospital Saturday collection at Colchester this year ; on the contrary, the amounts already received are almost equal to the total sum collected last year, and there are still a number of boxes from the camp and barracks to come in. This result is no doubt due to the energetic efforts of the Rev. J. W. Irvine and other members of the committee.

NOBLE'S Isle of Man Hospital is practically completed, and will shortly be handed over to the Hospital Committee. The building, which is a handsome structure, has been erected at the sole cost of Mr. and Mrs. H. B. Noble, and will be of invaluable service to the island generally.

THE outbreak of small-pox which has recently been causing so much alarm at Sheffield, is said to be perceptibly decreasing. Twenty convalescent patients were removed from the borough hospital a few days ago, and taken to the temporary building at Totley. Altogether there have been nearly seven hundred cases, and the deaths averaged about ten per cent. A very large proportion of those who have died were unvaccinated.

J. H. L., writing to the *St. James's Gazette*, strongly advises the employment of male nurses for male hospital patients. He considers that such nurses are especially required in many cases of brain diseases, in which it is impossible for female nurses to exercise adequate control over their patients.

MISS MARY WARDELL, of the Scarlet Fever Convalescent Home, writing to the *Times*, expresses the opinion that the large proportion of scarlet-fever cases during the present epidemic are of a mild type ; and considers that this is proved by the very low mortality which prevails, in spite of the overflowing condition of the fever hospitals. The proportion of those convalescing from severe attacks is said to be unusually small.

THE public are reminded that with the approach of shorter days and long evenings, interesting books and illustrated and other newspapers are much needed by hospital patients. Any congregation or other centre of influence might be used for the collection of such books and periodicals, and we commend the matter to those who bear office in religious or other societies.

THE managers of the National Dental Hospital and College, Great Portland Street, have resolved to admit ladies to be trained at their institution. This resolution is owing to the fact that although ladies have been able to obtain qualifications and licences entitling them to practise as dentists, the necessary education for enabling them to do so has been hitherto unprovided for in England. In 1876 the number of cases treated at the hospital was 8,509 ; in the short space of ten years the number had increased to 34,721. Steps are now being taken to enlarge the hospital buildings.

GREEN peas and other vegetables and fruit are cultivated at the Milton Fever Hospital, near Sittingbourne. Mr. Miller called attention to an item of £46 7s. 4d. in the account of the hospital, part of which, he understood, had been spent in the employment of a professional gardener, and in the cultivation of fruit and vegetables. He entered his most emphatic protest against what he called such silly proceedings. He considered that the sticks which were purchased for the training of the green peas cost more than the peas were worth. Perhaps Mr. Miller is one of those unhappy persons with whom green peas disagree, and fears the injurious effect on the stomachs of the patients.

THE following vacancies are announced this week, the amount of the salary being placed in each case after the name of the institution :—

**Resident Medical Officers.**

Birmingham General Dispensary.—£150, and £30 for cab hire.
Carnarvon and Anglesea Infirmary. House-Surgeon.—£100.
Ingham Infirmary, South Shields. Senior and Junior House-Surgeons.—£60 and £50.
Royal Hants Hospital, Winchester. House-Surgeon.—£100.

**Non-resident Medical Officers.**

Bristol Royal Infirmary. Obstetric Physician.
Taunton and Somerset Hospital. Honorary Surgeon.

**Matrons.**

Staffordshire General Infirmary.—£60.
Widnes Infectious Hospital. (Trained Nurse).—£30.

**Trained Nurses.**

Bristol Hospital for Sick Children. 1 Surgical; Medical.
Bristol Royal Infirmary. Several.—£16 to £18.
Companion Nurse (mental) for Lady, 2, Depoorie Villas, Anerly Road, Croydon.
Ealing Cottage Hospital. Under-Nurse.—£30.
Hampstead Home Hospital. Several.—From £20.
Kidderminster Fever Hospital.—£30.

**Probationers.**

Blackheath Cottage Hospital. One.
Bristol Royal Infirmary. Several.
City of Glasgow Fever Hospital. Several.—£18.
Newark Hospital, Notts. One.

---

WHAT may be considered as the most complete and perfect hospital with circular wards which has yet been erected, was opened at Hastings, Sussex, on the 13th inst. The Bishop of Winchester, Viscount Hampden, Mr. Noble, M.P., the Mayor, Dr. Bristowe, Mr. Timothy Holmes, Professor Marshall, and Mr. Burdett, were the chief speakers at the opening ceremony, and at the luncheon which followed.

---

THE architects of the Hastings Hospital, Messrs. Keith D. Young and Henry Hall, are to be congratulated upon a genuine success. From one end of the building to the other evidence is forthcoming to prove their acquaintance with the most recent and perfect appliances for hospital purposes which are known to the initiated. The furniture for the wards has been supplied by Messrs. Jenks and Wood, of 66, Berners Street, W., who have attained a high reputation for this class of work since they furnished Fitzroy House for The Home Hospitals Association, nearly ten years ago. This firm has made a study of hospital furniture, and several of their productions are as novel as they are useful. The bamboo screens in the wards at Hastings are, however, nine inches short of what they ought to be, to enable a patient confined to bed to be protected from the glare of the gas over the fire-places.

---

THERE are two or three small matters at the Hastings Hospital which might be improved. We have always advocated good, airy, and pleasant apartments for the resident staff in both hospitals and asylums. At Hastings, where the hospital contains some seventy beds at the outside, the matron has been given a sitting-room so spacious as to be out of all proportion to her requirements. This is to be regretted, as the children who ought to occupy this room have been relegated to a smaller room upstairs, where there are more beds than there ought to be. It is to be hoped the good-sense of the matron will induce her to propose that her present bedroom shall be made into a sitting-room, and the children restored to the airy and capacious ward which is now devoted to her sole use.

---

THE committee's attention ought to be directed to the way in which the walls of this new hospital have been dealt with by a barbarian, who has taken a number of brass-headed nails and hammered them into the walls on every floor, without any regard to propriety or purpose. In a building which is full of excellent work, and of which the builder, Mr. Holloway, has reason to be proud, it is lamentable to think that damage and disfigurement should have been permitted on the part of some workman, who has been sent in, we imagine without the knowledge of the architects, to put up bottle-racks, thermometers, hand-grenades, etc., in the wards and corridors. The wards contain twelve beds, and it is noticeable that the four single gas-brackets placed over the chimney-pieces give ample light, and that the gas pendants will only have to be lit when a serious case may require special attention for a length of time.

---

WE understand that a vacancy has occurred in the matronship of the Robinson Kay Branch of the Northern Counties Hospital for Incurables, in consequence of the resignation of the lady superintendent, Miss Bloomer, who has resigned her charge owing to her approaching marriage with Dr. Hamilton, of Bury, visiting surgeon to the hospital.

---

THE *Weymouth Telegraph* says :—" The invidious distinctions between Church clergymen and Nonconformist ministers which were made by the framers of the (Dorset County) Hospital rules, and who displayed thereby a sad lack of Christian charity, were denounced in no measured terms by Mr. Pearce-Edgecumbe, and if we form a true estimate of the qualities of the committee selected by him for the revision of the rules, one of their first acts will be to sweep them away altogether. Such distinctions, humiliating alike to minister and people, belong to a past age, and have always been a positive disgrace to the hospital. Churchmen and Dissenters meet on common ground inside the doors of such a house, and it is a very poor standard of Christianity which would devise barriers and distinctions there. For economic reasons, if for no higher, the rule should be amended. With a falling exchequer the hospital cannot afford to estrange dissenting subscribers, who have shortened their hand very considerably in the past, but who would, with the removal of the obnoxious anomalies which now exist, support the institution much more liberally." The *Telegraph* refers to the rule which allows the clergyman to visit a member of his congregation in hospital at any time, whereas the Nonconformist minister can only do so when he is specially sent for by any member of his flock. Such rules are now becoming universally obsolete.

---

THE Rev. Fairfax Goodall, chaplain to the Bristol Royal Infirmary, writes :—" You were kind enough to notice in your paper the lamented decease of the late Mr. W. C. Lysaght, Junior Resident Medical Officer of this institution. It is the wish of many persons that we should have, in this infirmary, some permanent memorial of his personal and professional character, and especially of the manner in which death came to him. We have decided to place a memorial window in the chapel. About one hundred pounds will be needed, towards which sum I am able to reckon with confidence upon forty. If you can see your way to kindly placing this matter before your readers, I shall be greatly obliged." We heartily sympathise with the desire on the part of officials and others connected with the Bristol Royal Infirmary to commemorate the untimely death of Mr. Lysaght. It is a graceful intention, and one well calculated to develop in young medical officers an increased regard for the important duties they have to perform. There should not be the slightest difficulty in raising the additional sixty pounds required for the carrying out of the object. We hope to hear within a week or two that the money has been obtained, and that a window of suitable design is in course of preparation.

The Hull Infirmary is about to become the recipient of a munificent reversionary bequest. The late Mr. Matthew Cressy Lee, solicitor, of Hull and Bridlington Quay, left a personal estate of nearly £46,000, the greater part of which will, after the decease of some aged persons, revert to the hospital.

In the large and rapidly increasing town of Dundee there is no adequate provision for the isolation of small-pox cases. In view of the recent outbreak of that disease at the contiguous city of Perth, it is highly desirable that such accommodation be provided without delay. Dr. Anderson, the Medical Officer of Health, recommends the immediate provision of such accommodation. The sanitary authorities will be well advised to give prompt attention to the subject.

The extraordinary scientific standpoint of Dr. Stirling, of Perth, is considered elsewhere. The matron of the Perth Hospital seems to be entirely at one with Dr. Stirling in his views about sanitary science. It is said that she visits the sick-wards of the Perth Infirmary where the small-pox cases are lying, and then, without changing her garments or using disinfectant precautions, sallies forth among the other nurses and patients. Dr. Simpson, the Medical Officer of Health for Perth, says that he is not greatly astonished at this, as the matron confessed to him that she did not know much of the principles of disinfection. If there are many hospital officials as ignorant as the Perth matron, and many infirmary directors as incapable of intelligent direction as those of Perth, it is time that some central authority were created for the better control and management of those important institutions.

"An Old Hospital Nurse" writes to make a complaint which has considerable justice in it, namely, that probationers sent from private nursing institutions to learn special departments of medical and surgical nursing at a hospital, are obliged to give part of the time they spend there to kitchen-work, with which in many cases they are already familiar, instead of devoting all their attention to the work they come to the hospital to study. As the time they have to remain there is limited, and the fees for their training are paid by the superintendent of the institution to which they belong, she considers it a pity that they should not give themselves wholly to the subject they wish to acquire a knowledge of; and we do not doubt that considerate matrons will spare these probationers work with which they are already acquainted, and which is unnecessary for them, though it may be exceedingly valuable for those nurses who are to receive all their training at the hospital, and will probably spend two or three years within its walls, with ample time and opportunity to learn every department of their work.

The Board of the Montrose Asylum had a merry meeting the other day. They originally intended to spend £10,000 upon new buildings. The architect, Mr. Sydney Mitchell preferred the sum of £15,000. The committee, however, decided, but not by resolution, that on no account should more than £13,000 be spent. The Rev. Mr. Fraser, who seems to be a highly economical clergyman, insisted upon going back to the £10,000, because there had been no resolution minuted or passed as to the £13,000. The Provost said it was perfectly well understood that £13,000 was to be the maximum, but if Mr. Fraser wished, the meeting would pass a vote of censure on itself for its former behaviour. Mr. Fraser, however, was of opinion that as the meeting was not in a penitent mood, the resolution would have little value. It was finally agreed that the sum of £13,000 should not be exceeded.

An outbreak of scarlet fever of mild type has occurred at Liverpool. Unless the epidemic should assume extraordinary proportions, it is believed that the present accommodation will be sufficient.

The Ilkeston Cottage Hospital, although it has only been in operation for three years, has managed to get into the condition, so common with hospitals, of being a little behind in its accounts. The expenditure for the year was £292 18s. 3d., whilst the income was only £251 5s. 9d. Both these amounts are very small for a town of such a considerable population as Ilkeston. There ought to be no difficulty whatever in raising twice as much for hospital purposes as the larger sum.

A few days ago a man was taken to the Oswestry Cottage Hospital with his throat cut, but was refused admission. Almost simultaneously a woman who had been in the hospital for some days was removed to the workhouse, although in a dying condition. These two incidents have excited much comment in the neighbourhood, and it may be hoped that the hospital authorities are prepared with a satisfactory explanation.

The managers of the Hartlepool Hospital have resolved that none but serious accident and other cases shall be treated at the institution. It appears to have been the custom to send all kinds of trifling injuries from factories to the hospital, even where the factories employ their own medical man. Such a course may be pleasant and profitable to factory doctors, but does not commend itself to hospital officials or subscribers.

A Hospital Sister writes:—"May I inquire, through your paper, what benefit would be derived from the proposed Pension Fund by those who have spent the best part of their lives in nursing the sick? According to the plans suggested, a nurse who enters the profession at twenty, and continues to work steadily till she is fifty years of age, would, in the end, be no better off than one who begins fifteen or twenty years later. Surely the rate of premium should be regulated by the number of years' service. I also wish to ask, in what way hospital officials, such as secretaries who have never undertaken the arduous duties of nursing, are entitled to a nurses' pension, or to any bounty given for that purpose? Why should highly-paid officials, who are able to save, derive more benefit from the Queen's goodness than hard-working, poor nurses, who, on account of the comparatively low wages given in all hospitals, would be unable to pay a large premium?"

Dr. Robertson, F.R.C.P., presided at the quarterly meeting of the committee of management of the Devonshire Hospital on Saturday. Over a thousand patients were admitted to the hospital during the three months, a much larger number than in any corresponding quarter in preceding years. Nearly all these patients were sent away improved. The bulk of the cases were of course of a rheumatic, gouty, or neuralgic character. Although the patients were more numerous the funds were considerably smaller, and the deficiency is principally due to the diminished amounts obtained by the collecting books in some of the hotels and lodging-houses of Buxton. Under this head alone the quarter's deficiency amounts to £77 6s. 10d. The offertories at the churches and chapels also show a considerable falling off. On the other hand, casual subscriptions and payments for patients have somewhat increased. The financial condition of the hospital is such as to occasion the managers a considerable degree of anxiety.

